# Clinical and pathological features of idiopathic membranous nephropathy with focal segmental sclerosis

**DOI:** 10.1186/s12882-019-1641-2

**Published:** 2019-12-16

**Authors:** Jiatong Li, Bing Chen, Caifeng Gao, Jing Huang, Yongmei Wang, Shiyin Zhang, Ying Xu, Wenkai Guo, Rong Wang

**Affiliations:** 1 Department of Nephrology, Shandong Provincial Hospital Affiliated to Shandong University, Jinan, Shandong Province 250021 People’s Republic of China; 2 Department of Geriatric, Shandong Provincial Hospital Affiliated to Shandong First Medical University & Shandong Academy of Medical Sciences, Jinan, Shandong Province 250021 People’s Republic of China; 3 Department of Nephrology, Jinan Shizhong People’s Hospital, Jinan, Shandong Province 250002 People’s Republic of China; 4 Department of Nephrology, Shandong Provincial Hospital Affiliated to Shandong First Medical University & Shandong Academy of Medical Sciences, Jinan, Shandong Province 250021 People’s Republic of China

**Keywords:** Idiopathic membranous nephropathy, Focal segmental sclerosis, Cyclophosphamide, Calmodulin inhibitor, Prognosis

## Abstract

**Background:**

The goal of this study was to investigate the clinical and pathological features and prognosis of idiopathic membranous nephropathy (IMN) with focal segmental lesions.

**Methods:**

In our hospital, 305 patients with nephrotic syndrome confirmed as IMN by renal biopsy were divided into a non-focal segmental lesion group (FSGS- group) and a focal segmental glomerulosclerosis (FSGS) group (FSGS+ group) and retrospectively analyzed. In all, 180 patients were followed for periods ranging from 6 months to 2 years. The general clinicopathological data of both groups were compared, and the effects of different treatment schemes on the prognosis of both groups were observed.

**Results:**

The FSGS+ group had a longer disease course, higher blood pressure levels, and higher serum creatinine and β_2_-microglobulin levels than did the FSGS- group (all *P* < 0.05). Pathologically, the FSGS+ group had increased glomerular sclerosis, glomerular mesangial hyperplasia, and acute and chronic tubular lesion rates (all *P* < 0.05). The remission rate was lower in the FSGS+ group than in the FSGS- group (64.7% vs 82.2%) and, among patients in the FSGS+ group, was lower in patients treated with calmodulin inhibitors than in those treated with cyclophosphamide (*P* < 0.01). Survival analysis showed that the FSGS+ group had a poor prognosis (χ^2^ = 4.377, *P* = 0.036), and risk factor analysis suggested that age at renal biopsy (*P* = 0.006), 24-h urinary protein quantity (*P* = 0.01), chronic tubulointerstitial lesions (*P* = 0.055), and FSGS lesions (*P* = 0.062) were risk factors for worsening renal condition; furthermore, 24-h urinary protein quantity was an independent risk factor for worsening renal condition.

**Conclusions:**

Membranous nephropathy with FSGS is a risk factor, but not an independent risk factor, for IMN. Patients with membranous nephropathy with FSGS often present hypertension and tubule injury. The nonselective drug cyclophosphamide is preferred, and calcineurin inhibitors should be used with caution.

## Background

Idiopathic membranous nephropathy (IMN) is the leading cause of adult primary nephrotic syndrome. Primary glomerular disease accounted for approximately 20% of IMN and is also the second or a third major cause of end-stage renal disease (ESRD) in primary glomerular nephritis patients [1, 2]. The pathological manifestations are characterized by the formation of immune complexes in the epithelial cells of the glomerular basement membrane and diffuse thickening of the glomerular basement membrane. The antigens of the glomerular podocytes and/or basement membrane form immune complexes to activate complements, resulting in damage to the filtration barrier, which in turn causes symptoms such as proteinuria [2, 3]. The natural course of the disease is long-lasting and variable, with approximately one-third of patients showing varying degrees of persistent proteinuria without disease progression and one-third of patients experiencing progressive deterioration in renal function. ESRD eventually develops in some patients, while other patients show spontaneous remission that can last for many years [4, 5]. Therefore, understanding the factors that affect the prognosis is critical for developing treatment plans.

Notably, previous studies have shown that sex, age, blood pressure, estimated glomerular filtration rate (eGFR), serum creatinine proteinuria, and urinary red blood cell count are prognostic factors, while other studies have shown that anti-phospholipase A2 receptor antibody is also an important biochemical indicator to help predict prognosis and guide treatment [5–10]. In recent years, nephrological changes, especially membranous nephropathy with focal segmental glomerulosclerosis (FSGS), cannot be ignored as a prognostic index in patients with IMN. However, recent research conclusions are not obvious; Chen Y et al. suggested that segmental sclerosis and tubulointerstitial injury rather than arteriosclerosis or C3 deposition are independent risk factors for ESRD in patients with membranous nephropathy [11]. Bazzi C et al. found that patients with IMN with high urinary N-acetyl-beta-glucosaminidase levels and low eGFRs were at an increased risk of progression [12]. Uchika Gupta et al. suggested that FSGS may be secondary to membranous nephropathy and may be an indication of poor prognosis [13]. However, Saskia F. Heeringa et al. suggested that FSGS is not an accurate predictor of renal outcome in patients with IMN and that renal biopsy results cannot be used to guide decisions on immunosuppressive therapy [14]. The different views of these people suggest that the predictive effect of FSGS and other factors on the prognosis of membranous nephropathy is controversial and needs to be further studied.

We also found that approximately one-fifth of patients with membranous nephropathy complicated with FSGS lesions (IMN-FSGS) were found during renal biopsy. In this paper, the clinicopathological features of cases of IMN-FSGS were analyzed. Then, through follow-up, the remission rates of several common clinical regimens were compared, and some suggestions for the treatment of membranous nephropathy complicated with focal segmental sclerosis were proposed. Finally, the risk factors affecting the prognosis of membranous nephropathy were further analyzed.

## Methods

### Patient selection

From January 2015 to December 2017, 305 adult patients at Shandong Provincial Hospital who were diagnosed with nephrotic syndrome confirmed by renal biopsy as IMN and had complete clinical data were included in this study. All the patients had IMN; this condition is universally diagnosed by kidney biopsy through the presence of subepithelial spikes along capillary walls on silver staining, granular IgG and C3 along capillary walls on immunofluorescence, and subepithelial deposits on electron microscopy (EM). However, those with secondary membranous nephropathy caused by systemic lupus erythematosus (SLE), hepatitis B, heavy metal poisoning, other infections or malignancy were excluded. At the time of selection, the patients’ renal function was in the normal range. A total of 180 patients had complete follow-up data for periods ranging from 6 months to 2 years. This study was approved by the Ethics Committee of Shandong Provincial Hospital.

### Clinical and laboratory data

#### General information

Sex, age, course of disease, and systolic and diastolic blood pressure were taken at the time of biopsy.

#### Laboratory examination

Kidney damage indicators included peripheral blood leukocyte, hemoglobin, and platelet counts as well as glutamic pyruvic transaminase (ALT) and glutamic oxaloacetic transaminase (AST), serum total protein (TP), albumin (ALB), globulin (GLB), blood retinol-binding protein, beta 2-microglobulin, superoxide dismutase (SOD), cystatin C, serum creatinine, urea nitrogen, blood lipid, blood glucose, and blood calcium levels. Routine blood tests evaluated hematuria, proteinuria, and 24-h urinary protein quantity. Immunological indicators included serum IgG, IgA, and IgM levels; serum complement (C3 and C4) levels; and the presence of antinuclear antibodies (ANAs) and antineutrophil cytoplasmic antibodies (ANCAs). An ELISA was used to detect the antibody levels of phospholipase A2 receptors (PLA2R) in the serum of patients. Anti-PLA2R ELISA (IgG) kits were purchased from EUROIMMUN Medizinische Labordiagnostika AG. The results were considered negative a t < 20 relative units (RU)/mL and positive at ≥20 RU/mL. The eGFR was calculated with a modified Modification of Diet in Renal Disease (MDRD) formula as follows: eGFR [ml/min/1.73 m2] = 186 × [Scr (μmol/L)/88.4]-1.154 × age-0.203(× 0.742 if female).

### Pathological data

Each patient’s renal biopsy included at least 10 glomeruli for histopathological evaluation. Renal puncture tissue was examined by light microscopy, immunofluorescence and electron microscopy. Two renal pathologists, Dr. JZ and Dr. YX, participated in the reading and review of pathological results. Dr. JZ was responsible for the biopsy and preliminary reading of the renal pathology, and Dr. XY was responsible for the re-review of the pathological results. Membranous nephropathy was divided into 4 stages. If two stages were noted at the same time, the relatively higher stage was selected; stages III and IV were defined as advanced pathological stages. Glomeruli were observed for complications involving focal segmental sclerosis, glomerular sclerosis, mesangial proliferative lesions, crescents, endothelial hyperplasia, acute tubular lesions, chronic tubular lesions, inflammatory cell infiltration, small vessel lesions, and so on. FSGS lesions were defined as having focal and segmented distributions of glomerular lesions under light microscopy; they mainly manifested with an increased mesangial matrix and balloon adhesion, accompanied by a small amount of mesangial hyperplasia and corresponding tubular atrophy and renal interstitial fibrosis. The incidence of spherical sclerosis was defined as the existence of spherules in the pathological section. A glomerular mesangial proliferative lesion was defined as the number of cells in the mesangial region greater than or equal to 4. Glomerular endothelial hyperplasia was defined as observable endothelial hyperplasia. Crescent incidence was defined as the presence of cellular or fibrous crescents in one of the glomeruli. The incidence of acute tubulointerstitial lesions was defined as at least 10% renal tubular epithelial flattening, brush edge exfoliation and necrosis, and nontubular inflammatory cell infiltration in the atrophy zone. Chronic renal tubulointerstitial lesions were defined as more than 10% renal tubular atrophy and interstitial fibrosis. Interstitial inflammatory lesions were characterized by neutrophil infiltration and small vascular lesions due to arterial intimal thickening, elastic layer stratification and hyaline degeneration.

### Follow-up records

The clinical assessment of the treatments was evaluated by changes in proteinuria and serum creatinine. The nephrotic range for proteinuria was defined as a 24-h urine T*P* value ≥3.5 g. Complete remission (CR) was defined as urine protein < 0.3 g/d in a previously nephrotic patient. Partial remission (PR) was defined as a 50% reduction in the urine protein to a level of proteinuria < 3.5 g/d. We defined worsening renal condition as doubling of the baseline Scr level; we defined end-stage renal disease (ESRD) as a creatinine clearance rate of less than 15 ml/min at last follow-up, start of dialysis or renal transplantation. Serious complications were clinical death or severe pulmonary infection, pulmonary embolism, cerebral infarction, myocardial infarction, tumor and other diseases.

#### Statistical analyses

SPSS 19.0 statistical software was used for data analysis. Quantitative variables with normal distributions were expressed as x ± s and compared by t-tests, and data with abnormal distributions were expressed as medians and compared by nonparametric test. The categorical variables were expressed as rates and were compared by the χ^2^ test. Patient and renal survival were estimated by the Kaplan-Meier method. The relationships of the covariates to patient and renal survival were evaluated in the univariate analysis with the log-rank test and in the multivariate analysis with the Cox proportional hazards model; the Harrel C statistic was used for verification of the risk factors confirmed by the Cox proportional hazards model. A *P* value <.05 was considered statistically significant, and a *P* value < 0.01 was considered notably statistically significant. A Cox analysis of risk factors was performed in combination with clinical significance, and *P* < 0.10 was considered statistically significant.

## Results

### General clinical data

Approximately 252 patients (82.6%) in the IMN without focal segmental sclerosis of the glomeruli group (FSGS- group) and 53 patients (17.4%) in the membranous nephropathy complicated with focal segmental sclerosis of the glomeruli group (FSGS+ group) were included in this study; the general information of the patients is shown in Table 1. The average course of disease in the FSGS+ group was 5.33 ± 5.93 months, which was longer than that in the FSGS- group, and the difference was statistically significant (*P* < 0.05). Patients in the FSGS+ group were slightly older than those in the FSGS- group, but the difference was not statistically significant (*P* = 0.195). The baseline hypertension rate in the FSGS+ group was 75.5%, which was higher than 46.0% in the FSGS- group (*P* < 0.01), and the mean systolic and diastolic blood pressures in the FSGS+ group was higher than those in the FSGS- group (*P* < 0.05). The mean 24-h urinary protein quantity in the FSGS+ group (6.18 ± 3.19) was slightly higher than that in the FSGS- group (5.60 ± 3.07), but there was no significant difference (*P* = 0.227). The levels of serum creatinine (70.28 ± 20.25 vs 64.21 ± 18.64), β2-microglobulin (2.63 ± 1.12 vs 2.15 ± 0.66) and retinol-binding protein (56.74 ± 17.55 vs 51.11 ± 16.52) in the FSGS+ group were higher than those in the FSGS- group. The difference was statistically significant (*P* < 0.05). In addition, we found that platelet counts and superoxide dismutase (SOD) and serum IgG4 levels in the FSGS+ group were significantly higher than those in the FSGS- group (*P* < 0.05). The total positive rate of antiphospholipase A2 receptor antibodies in the FSGS- group was similar to that in the FSGS group.

**Table 1:** Clinical and Laboratory Findings at Time of Renal Biopsy

	IMN(*N* = 305)	FSGS-(*N* = 252)	FSGS+(*N* = 53)	*P* value
Age (year)	44.23 ± 13.95	43.62 ± 13.61	46.53 ± 15.09	0.195
Sex (female(%))	114/305 (37.4)	96/252 (38.1)	18/53 (33.9)	0.572
Duration of illness (month)	3.87 ± 4.68	3.46 ± 4.20	5.33 ± 5.93^*^	0.028
SBP (mmHg)	141.57 ± 19.98	138.03 ± 19.28	152.50 ± 18.46^**^	0.003
DBP (mmHg)	87.52 ± 13.24	85.68 ± 12.46	93.23 ± 14.23^*^	0.019
Urine RBC/HPF	13.92 ± 16.72	13.59 ± 15.98	15.18 ± 19.45	0.557
24-HUPRO (g/L)	5.71 ± 3.09	5.60 ± 3.07	6.18 ± 3.19	0.227
WBC (× 10^9^/L)	7.64 ± 3.27	7.61 ± 3.14	7.73 ± 3.77	0.831
HGB (g/L)	136.12 ± 17.36	136.40 ± 17.31	134.82 ± 17.74	0.604
PLT (×10^9^/L)	260.06 ± 61.47	254.64 ± 58.81	280.85 ± 67.45^**^	0.008
AST (u/L)	23.06 ± 14.14	21.98 ± 10.32	27.16 ± 23.24	0.138
ALT (u/L)	25.55 ± 22.18	24.46 ± 17.49	29.70 ± 34.53	0.145
SOD (u/mL)	96.36 ± 29.21	92.33 ± 26.43	110.57 ± 34.19^**^	0.005
TP (g/L)	45.58 ± 7.20	45.37 ± 6.78	46.34 ± 8.67	0.408
ALB (g/L)	23.82 ± 5.26	23.77 ± 5.09	24.05 ± 6.05	0.723
GLO (g/L)	21.85 ± 4.59	21.75 ± 4.30	22.19 ± 5.56	0.554
GLU (mmol/L)	5.22 ± 1.41	5.24 ± 1.47	5.12 ± 1.05	0.628
BUN (mmol/L)	5.45 ± 1.91	5.44 ± 1.86	5.49 ± 2.11	0.873
SCr (μmol/L)	66.26 ± 19.03	64.21 ± 18.64	70.28 ± 20.25^*^	0.036
CysC (mg/L)	1.05 ± 0.29	1.05 ± 0.28	1.06 ± 0.33	0.745
β2-MG (mg/L)	2.25 ± 0.80	2.15 ± 0.66	2.63 ± 1.12^**^	0.010
RBP (mg/L)	52.32 ± 16.87	51.11 ± 16.52	56.74 ± 17.55^*^	0.047
Ca (mmol/L)	2.16 ± 0.26	2.15 ± 0.27	2.18 ± 0.21	0.468
CHOL (mmol/L)	8.85 ± 2.72	8.91 ± 2.76	8.63 ± 2.59	0.551
HDL-C (mmol/L)	1.74 ± 0.59	1.75 ± 0.58	1.74 ± 0.62	0.930
LDL-C (mmol/L)	5.33 ± 2.18	5.38 ± 2.19	5.14 ± 2.11	0.531
PLA2R (POSITIVE %)	132/175 (75.4)	98/132 (74.2)	34/43 (79.0)	0.523
IgG4 (mg/L)	313.08 ± 248.47	283.81 ± 223.11	380.84 ± 291.16^*^	0.044
IgG (g/L)	5.28 ± 2.53	5.23 ± 2.47	5.50 ± 2.81	0.547
IgM (g/L)	1.09 ± 0.54	1.11 ± 0.56	0.99 ± 0.42	0.191
IgA (g/L)	2.20 ± 0.87	2.19 ± 0.79	2.22 ± 1.14	0.912
C3 (g/L)	1.16 ± 0.25	1.16 ± 0.25	1.17 ± 0.25	0.804
C4 (g/L)	0.29 ± 0.08	0.28 ± 0.08	0.29 ± 0.07	0.528
C1q (mg/L)	207.13 ± 41.49	206.48 ± 40.21	210.11 ± 47.18	0.612

Note: *SBP* systolic blood pressure, *DBP* diastolic blood pressure, *24-HUPRO* 24-h urinary protein, *WBC* white blood cell, *HGB* hemoglobin, *TP* serum total protein, *ALB* serum albumin, *BUN* blood urea nitrogen, *SCr* serum creatinine concentration, *SOD* superoxide dismutase, *CysC* cystatin C, *RBP* blood retinol-binding protein, *β2-MG* β2-microglobulin, *BUN* blood urea nitrogen, *ALT* glutamic pyruvic transaminase, *AST* glutamic oxaloacetic transaminase, *GLB* globulin, *Ca* blood calcium, *C3 and C4* serum complement3 and 4, *PLA2R* phospholipase A2 receptors

Compared with the FSGS- group and the FSGS+ group, P* < 0.05, P** < 0.01

### Pathological data

In this study, the pathological stages of membranous nephropathy in the FSGS+ group and FSGS- group were mainly stage I and stage II. Immunofluorescence examination of renal biopsy tissue showed that there was no significant difference in the deposition of IgG, IgM, IgA, C3 and C1q between the two groups, but the deposition intensity of fibrous tissue in the FSGS+ group was slightly higher than that in the FSGS- group (*P* < 0.05). The proportions of glomerular sclerosis, glomerular mesangial hyperplasia and glomerular endothelial hyperplasia in the FSGS+ group were significantly higher than those in the FSGS- group (*P* < 0.05). The proportions of acute renal tubulointerstitial lesions and chronic renal tubulointerstitial lesions in FSGS +group were also higher than those in the FSGS- group (*P* < 0.05). However, the proportion of acute and chronic tubulointerstitial lesions was less than 25%. in all patients. We also observed that the infiltration rate of interstitial inflammatory cells in the FSGS+ group was higher than that in the FSGS- group, but the difference was not statistically significant. The proportion of renal arteriolar lesions in the FSGS+ group was higher than that in the FSGS- group (*P* < 0.01). The renal pathology of patients is shown in Table 2.

**Table 2:** Histopathologic Parameters at the Time of Renal Biopsy

	FSGS-(*N* = 252)	FSGS+(*N* = 53)	*P* value
Pathological stage(%)			
I	136/252 (53.9)	23/53 (43.4)	0.161
II	114/252 (45.3)	30/53 (56.6)	0.132
III + IV	2/252 (0.8)	0	
Tissue IgG	2.77 ± 0.63	2.63 ± 0.82	0.370
Tissue IgM	0.45 ± 0.81	0.43 ± 0.75	0.967
Tissue IgA	2.30 ± 0.81	2.13 ± 1.36	0.597
Tissue C_3_	1.47 ± 1.08	1.31 ± 1.00	0.364
Tissue Fib	0.53 ± 1.22	0.13 ± 048^*^	0.025
Tissue C1q	0.80 ± 1.17	0.53 ± 0.77	0.146
Pathological characteristics			
Glomerular sclerosis	105/252 (40.5.)	30/53 (56.6)^*^	0.047
Mesangial proliferative lesions	67/252 (26.6)	30/53 (56.6)^**^	0.000
Crescents	2/252 (0.8)	1/53 (1.8)	0.464
Endothelial hyperplasia	5/252 (1.9)	6/53 (11.3)^**^	0.001
Acute tubular lesions	12/252 (4.7)	7/53 (13.2)^*^	0.021
Chronic tubular lesions	15/252 (5.9)	17/53 (32.0)^**^	0.000
Inflammatory cell infiltration	8/252 (3.2)	4/53 (7.5)	0.137
Small vessel lesions	57/252 (22.6)	23/53 (43.4)^**^	0.002

Note: Compared with the FSGS- group and the FSGS+ group, P* < 0.05, P** < 0.01

### Treatments and outcomes

Among the 305 patients, 180 patients were followed for more than 6 months, with an average follow-up time of 12.15 ± 5.27 months. There were 146 patients in the FSGS- group and 34 patients in the FSGS+ group. The patients received specific treatments, including glucocorticoids plus immunosuppressive agents (cyclophosphamide, cyclosporine A, tacrolimus). Our treatment plans were as follows: Patients on drug regimens were treated according to blood pressure and blood lipids. Acetylcholinesterase inhibitors (ACEIs) and angiotensin type 1 receptor blockers (ARBs) were used to control blood pressure below 140/90 mmHg, and statins were used to control blood lipids. Depending on the presence of edema, diuretics were sometimes used. Tacrolimus was administered according to the following regimen: the initial oral dose of tacrolimus was 0.5 mg/(kg·d), and the treatment was continued for at least 6 months. The plasma concentration of tacrolimus was determined for 1 week to maintain the plasma concentration of tacrolimus at 5~10 ng/ml. Cyclosporine A was administered according to the following regimen: the initial oral dose of cyclosporine A was 3–5 mg/(kg·d), and the treatment was continued for at least 6 months. The plasma concentration of cyclosporine A was monitored for 1 week to maintain the plasma concentration of cyclosporine A at 100~200 ng/ml. Cyclophosphamide was administered according to the following regimen: the drug was administered at a static dose of 750 mg/m^2^ body surface area for at least 6 months, and the cumulative dose was 6–8 g. All patients were given a sufficient dose of prednisone 1 mg/(kg·d). After 8 weeks of adequate treatment, the dose size was reduced by 5 mg every 2 weeks and then held constant at a low dose of 10 mg/d. The total course of treatment was at least 9 months. The overall response rate (including the CR and PR) in the FSGS+ group was 64.7%, which was significantly lower than that in the FSGS- group (82.2%), and the difference was statistically significant (*P* = 0.024). The CR rate and PR rate were 23.5 and 41.2% in the FSGS+ group and 36.3 and 45.9% in the FSGS- group, respectively. Further subgroup analysis showed that there was no significant difference in the remission rate between the two groups treated with sufficient corticosteroids and cyclophosphamide at the same time. The overall remission rate in the FSGS+ group was significantly lower than that in the FSGS- group (50.0% vs 86.2%, *P* < 0.01). See Table 3 for details. We performed subgroup analysis of the PLA2R- group and the PLA2R+ group. The remission rate (including CR and PR) in the PLA2R+ group was 75.4%, which was significantly lower than that in the PLA2R- group (89.7%). In the PLA2R+ group, the remission rate decreased as the PLA2R titer increased, especially in the high-titer group; the overall remission rate was 56.7%. The total remission rate in the high-titer group was significantly lower than that in the PLA2R+ group. However, in the PLA2R- group and the PLA2R+ group, there was no significant difference in the remission rate between the FSGS+ group and the FSGS- group, between the PLA2R+ group and the FSGS- group, or between the FSGS+ group and the FSGS- group. In short, the remission rate was not related to FSGS+ but was significantly related to the titer of PLA2R. For more information, see Table 4. Consistent with the above results, our results showed that the total 24- h proteinuria level in the FSGS+ group was significantly higher than that in the FSGS- group after treatment, and the difference was statistically significant (*P* < 0.05). The serum creatinine level in the FSGS+ group was significantly higher than that in the FSGS- group after treatment (*P* < 0.05). The subgroup analysis showed that the total 24-h proteinuria levels in the FSGS group were significantly higher than those in the FSGS- group after treatment (*P* < 0.05), mainly in patients with half-dose corticosteroids plus cyclosporine or tacrolimus (*P* < 0.05). The serum creatinine level in the FSGS group was significantly higher than that in the FSGS- group after tacrolimus treatment (*P* < 0.05). See Table 5 for details.

**Table 3:** Remission rate after 1 year of follow-up treatment

Total	CR	PR	CR + PR
MN(*N* = 180)	63/180 (35.0%)	79/180 (43.9%)	142/180 (78.9%)
FSGS-(*N* = 146)	53/146 (36.3)	67/146 (45.9)	120/146 (82.2)^*^
FSGS+(*N* = 34)	10/34 (29.4)	12/34 (35.3)	22/34 (64.7)
Pred + CTX			
FSGS-(*N* = 81)	33/81 (40.7)	31/81 (38.3)	64/81 (79.0)
FSGS+(*N* = 18)	8/18 (44.4)	6/18 (33.3)	14/18 (77.7)
Pred + CsA/ TAC			
FSGS-(*N* = 65)	20/65 (30.8)	36/65 (55.4)	56/65 (86.2)^**^
FSGS+(*N* = 16)	2/16 (12.5)	6/16 (37.5)	8/16 (50.0)
Pred + CsA			
FSGS-(*N* = 34)	11/34 (32.3)	16/34 (47.1)	27/34 (79.4)
FSGS+(*N* = 9)	1/9 (11.1)	4/9 (44.4)	5/9 (55.5)
Pred + TAC			
FSGS-(*N* = 31)	9/31 (29.0)	20/31 (64.5)	29/31 (93.5)^*^
FSGS+(*N* = 7)	1/7 (14.3)	2/7 (18.6)	3/7 (42.8)

Note: *CR* complete remission, *PR* partial remission, *CsA* cyclosporin, *Pred* prednisone, *TAC* tacrolimus, *CTX* cyclophosphamide, *MN* membranous nephropathy, *FSGS-* membranous nephropathy without focal segmental glomerulosclerosis, *FSGS+* membranous nephropathy with focal segmental glomerulosclerosis

Compared with the FSGS- group and the FSGS+ group, P* < 0.05, P** < 0.01

**Table 4:** Remission rate after 1 year of follow-up treatment between PLA2R+ and PLA2R- group

Total	CR	PR	CR + PR
PLA2R-	13/29 (44.8%)	13/29 (44.8%)	26/29 (89.7%)
FSGS- (*N* = 27)	12/27 (44.4%)	12/27 (44.4%)	24/27 (88.9%)
FSGS+ (*N* = 2)	1/2 (50.0%)	1/2 (50.0%)	2/2 (100%)
PLA2R+ FSGS- (*N* = 100) FSGS+(*N* = 18)	33/118 (27.9%) 28/100 (28.0%) 5/18 (27.8%)	56/118 (47.5%) 47/100 (47.0%) 9/18 (50.0%)	89/118 (75.4%) 75/102 (73.5%) 14/18 (77.8%)
Low titer PLA2R+(14–86)			
FSGS - (*N* = 47)	19/47 (40.4%)	20/47 (42.6%)	39/47 (82.9%)
FSGS + (*N* = 9)	3/9 (33.3%)	4/9 (44.4%)	7/9 (77.8%)
Low titer PLA2R+(87–204)			
FSGS- (*N* = 21)	4/21 (19.05%)	14/21 (66.7%)	18/21 (85.7%)
FSGS + (*N* = 4)	1/4 (25.0%)	2/4 (50.0%)	3/4 (75.0%)
High titer PLA2R+(> 204)			
FSGS - (*N* = 32)	6/32 (18.7%)	12/32 (37.5%)	18/32 (56.3%)
FSGS + (*N* = 5)	1/5 (20.0%)	2/5 (40.0%)	3/5 (60.0%)

Note: *CR* Complete remission, *PR* Partial remission, *FSGS-* membranous nephropathy without focal segmental glomerulosclerosis, *FSGS+* membranous nephropathy with focal segmental glomerulosclerosis, *PLA2R* phospholipase A2 receptors

**Table 5:** Data after 1 year of follow-up treatment

Total	24-HUPRO(g/L)	ALB(g/L)	SCr (μmol/L)	BUN (mmol/L)	New DM(%)
FSGS-(*N* = 146)	1.27 ± 1.58	36.82 ± 5.75	63.89 ± 17.05	1.49 ± 2.20	22/146 (15.1)
FSGS(*N* = 34)	2.18 ± 2.34^*^	34.74 ± 8.86	76.99 ± 35.05^*^	2.74 ± 2.71	4/34 (11.8)
Pred + Ctx					
FSGS-(*N* = 81)	1.37 ± 1.83	36.22 ± 5.94	59.36 ± 13.46	5.30 ± 1.59	11/81 (13.6)
FSGS(*N* = 18)	1.44 ± 1.95	35.16 ± 8.77	72.55 ± 43.73	7.33 ± 2.88^**^	2/18 (11.1)
Ped + CsA					
FSGS-(*N* = 34)	1.11 ± 1.24	37.68 ± 5.90	72.50 ± 21.68	6.17 ± 1.60	5/34 (14.7)
FSGS+(*N* = 9)	2.89 ± 2.81^*^	34.28 ± 7.59	77.74 ± 12.67	7.25 ± 3.14	2/9 (11.1)
Ped + TAC					
FSGS-(*N* = 31)	1.15 ± 1.11	37.46 ± 4.93	67.08 ± 16.16	6.21 ± 1.80	6/31 (19.4)
FSGS+ (*N* = 7)	2.71 ± 2.49^*^	35.07 ± 11.80	87.00 ± 31.15^*^	6.49 ± 1.69	0

Note: *ALB* serum albumin, *BUN* blood urea nitrogen, *SCr* serum creatinine concentration, *eGFR* estimated Glomerular filtration rate, *DM* diabetes

Compared with the FSGS- group and the FSGS+ group, P* < 0.05, P** < 0.01

### Prognosis analysis

**Fig. 1:**
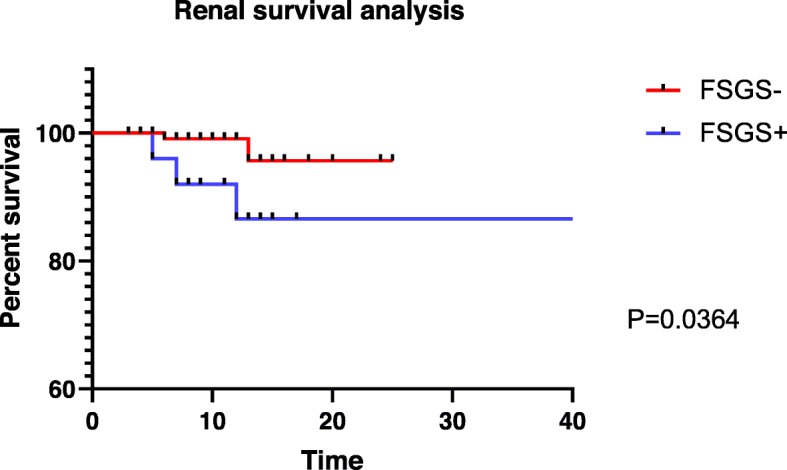
FSGS can predict renal outcome in patients with iMN. Note: See methods for definition of worsening renal condition. Renal survival is depicted for patients with FSGS (FSGS-, red line; *n* = 146) and without FSGS (FSGS+, blue line; *n* = 34)

At the end of the follow-up, 5 cases of worsening renal condition were identified, including 2 cases in the FSGS+ group and 3 cases in the FSGS- group. Four cases occurred in the corticosteroids plus cyclosporine or tacrolimus treatment group. The main reason for this result may be the high concentration of cyclosporine or tacrolimus; 2 patients presented negative proteinuria and 2 cases did not experience remission. Another case occurred in the corticosteroids plus cyclophosphamide group with the PR of proteinuria; the specific reasons were unknown. During follow-up, severe complications were observed in 3 patients; severe pneumonia occurred in 2 patients (1 death but no worsening renal condition) and intracranial fungal infection occurred in 1 patient. See Table 6 for details. Using a Kaplan-Meier survival plot, the log-rank test found that the 2 groups had significant renal survival differences (χ^2^ = 4.377, *P* = 0.036). The 2-year renal survival rate was 88.0% in the FSGS+ group and 98.5% in the FSGS group, as shown in Fig. 1. The Cox univariate analysis showed that the variables associated with worsening renal condition included age at the time of renal biopsy, 24-h urinary protein quantification, chronic tubulointerstitial lesions and FSGS lesions, as shown in Table 7. A Cox proportional hazard model was used to analyze age; 24-h urine protein, β2-microglobulin, and cystatin levels; chronic tubulointerstitial lesions and FSGS lesions at the time of renal biopsy. Twenty-four-hour urinary protein was found to be an independent risk factor for worsening renal condition, as shown in Table 8.

**Table 6:** Prognosis of patients in the two groups

Prognosis	FSGS-(*N* = 146)	FSGS+(*N* = 34)
Renal worsening condition	2	3
Clinical death	0	1
Severe infection	2	1
Complete remission	53	10
Partial remission	67	12

Note: *FSGS-* membranous nephropathy without focal segmental glomerulosclerosis, *FSGS+* membranous nephropathy with focal segmental glomerulosclerosis

**Table 7:** Univariate Cox regression analysis of the risk of worsening renal condition in patients with IMN

	B	SE	Walder	DF	Sig	Exp(B)
Age	.158	.058	7.520	1	.006	1.172
24-HUPRO	.262	.102	6.611	1	.010	1.300
Cys C	2.514	.931	7.295	1	.007	12.356
β_2_-MG	.839	.290	8.379	1	.004	2.314
FSGS	−1.714	.919	3.477	1	.062	.180
CTL	−1.609	.914	3.101	1	.078	.200
SVL	2.150	1.122	3.671	1	.055	8.581

Note: *CTL* Chronic tubular lesions, *SVL* Small vessel lesions, *24-HUPRO* 24-h urinary protein, *Cys C* cystatin c, *β2-MG* β2-microglobulin, *DF* degree of freedom, *Sig* significance, *FSGS* membranous nephropathy with focal segmental glomerulosclerosis

**Table 8:** Multivariate Cox regression analysis of worsening renal condition risk in patients with IMN

	B	SE	Walder	DF	Sig	Exp(B)
Age	.100	.070	2.045	1	.153	1.105
24-HUPRO	.302	.145	4.309	1	.038	1.352
Cys C	1.777	1.843	.929	1	.335	5.911
β_2_-MG	.330	.586	.318	1	.573	1.391
FSGS	−.955	1.302	.539	1	.463	.385
CTL	.909	1.344	.457	1	.499	2.481
SVL	−1.043	1.563	.446	1	.504	.352

Note: *CTL* Chronic tubular lesions, *SVL* Small vessel lesions, *24-HUPRO* 24-h urinary protein, *Cys C* cystatin c, *β2-MG* β2-microglobulin, *DF* degree of freedom, *Sig* significance, *FSGS* membranous nephropathy with focal segmental glomerulosclerosis

## Discussion

In recent years, a number of studies have reported the effect of lesions on the clinical characteristics and renal prognosis of IMN patients with FSGS, but the conclusions varied. Some studies have shown that the incidence of FSGS in IMN patients is between 12.8 and 43% [7, 8, 13], which is to our incidence of 17%. Dumoulin A et al. found that patients with FSGS had an increased degree of hypertension and an increased rate of severe renal damage [7]. Qiu-hua Gu et al. suggested that hypertension, abnormal serum creatinine levels, microscopic hematuria incidence and 24-h urinary protein excretion were higher in the FSGS+ group than those in the FSGS- group [15]. Some of these results were the same as ours, and some were different. Our analysis shows that the main reason was the degree of severity of the patients involved in the group. The renal functions of the patients we enrolled were in the normal range, and the pathological injuries were relatively minor, so the hematuria and renal injuries were relatively minor.

In terms of pathology, we observed that the proportions of FSGS glomerulosclerosis, glomerular mesangial hyperplasia, glomerular endothelial hyperplasia and renal arteriolar mucoid degeneration in the FSGS+ group were significantly higher than those in the FSGS- group, and the proportions of acute tubular lesions and chronic tubular lesions were also higher than those in the FSGS- group. The Gupta R study showed that patients with FSGS had more severe renal lesions, a higher mean staging of membranous lesions, more nonfunctional glomeruli, more severe mesangial hyperplasia, and more severe interstitial fibrosis and vascular changes than those without FSGS [13]. Qiu-hua Gu et al. showed that renal tubular atrophy, interstitial infiltration and were relatively common and severe [15]. The above studies were basically consistent with our observations, but we relaxed the standard for chronic tubular injury to 10% (the proportion of chronic tubular injury in all patients was not more than 25%). This change was made because we found that 10% of renal tubular injuries indicate a poor prognosis, so we reduced the percentage. For treatments and outcomes, the patients we selected were not given nonspecific treatment and observed for 6 months but rather were given specific treatment directly because the average daily urinary protein of the patients was more than 6 g. These patients had at least a moderate or high risk of progressive disease. In addition, CR had a better long-term prognosis, and PR reduced the risk of renal failure [16, 17]. A recent study showed that after IMN patients achieved CR or PR, the risk of renal function deterioration was significantly reduced [18]. For IMN patients with severe proteinuria, early treatment may be an effective method. We found that the overall response rate of the FSGS+ group was 64.7%, which was significantly lower than that of the FSGS- group (82.2%). Further subgroup analysis showed that there was no difference between the two groups when using the corticosteroids plus cyclophosphamide regimen, but the remission rate associated with cyclosporine and tacrolimus in the FSGS+ group was lower than that in the control group. According to our analysis, patients with membranous nephropathy complicated with FSGS had high levels of proteinuria, most patients had chronic tubular interstitial lesions, and the kidneys themselves has ischemic manifestations. Cyclosporine and tacrolimus are calmodulin neurophosphatase inhibitors (CNIs), and their mechanism is similar. One stabilizes the cytoskeleton of podocytes by inhibiting the expression of transient receptor potential cation channel 6 (TRPC6) proteins and calmodulin neurophosphatase (CaN), thereby reducing urinary protein [19, 20]. The other inhibits the translocation of nuclear factors in activated T cells (NFAT) by binding to calmodulin neurophosphatase, thus blocking the transcription of early T lymphocyte activation genes induced by T cell receptor (TCR)-CD3, and blocking the transition of T cells from the G0 phase to the G1 phase [21]. It has been reported that excessive doses of cyclosporine can lead to increased renal vascular resistance, especially glomerular afferent arterioles, resulting in renal ischemia. This might be the mechanism responsible for chronic tubular interstitial and vascular changes associated with chronic calcineurin toxicity [22]. However, nephrotoxicity associated with CNIS is an important issue of concern, limiting its clinical application. In this study, we observed a significant increase in serum creatinine during CNIS therapy. We speculated that the patients with membranous nephropathy with focal segmental sclerosis had chronic tubular interstitial injury. In these patients, the long-term use of cyclosporine and tacrolimus may further reduce the original glomerular blood supply and further aggravate chronic injury of the tubulointerstitial tissue, especially in patients with high blood concentrations, so the remission rate is lower than that of the cyclophosphamide group. Therefore, cyclosporine and tacrolimus should be used with caution in such patients, and their side effects should be closely observed.

The survival curve showed that the renal survival rate of the FSGS+ group was lower than that of the FSGS- group. Risk factor analysis revealed that age at renal biopsy; 24-h urinary protein, β_2_-microglobulin, and cystatin levels; chronic tubulointerstitial disease; and complication with FSGS were all risk factors for IMN. According to the Cox proportional hazard model, only 24-h urinary protein was an independent risk factor for worsening renal condition. The univariate Cox analysis by Ke Zuo et al. showed that as eGFR decreased, proteinuria, hypertension, the N-acetyl-β-D-glucosidase (u-NAG) level and the retinol-binding protein (u-RBP) level increased in the nephrotic system. Tubular interstitial disease and renal arteriopathy are risk factors for renal survival, but total/segmental sclerosis is not a risk factor. The Cox multivariate analysis showed that decreased eGFR and chronic tubulointerstitial damage were independent risk factors for end-stage renal disease (ESRD) [10]. Shiiki H et al. suggested that tubulointerstitial lesions are an important predictor of the progression of nephrotic syndrome to end-stage nephropathy [10]. Dumoulin A and Gupta, R. et al. considered chronic tubulointerstitial damage as an independent risk factor for ESRD and that the presence of focal segmental glomerulosclerosis was the most important prognostic factor [7, 13]. This was similar to the results of our study. Although membranous nephropathy with segmental sclerosis was a univariate predictor of ESRD, it was not an independent risk factor in the multivariate analysis. The limitations of this study were that is was a retrospective observational study, and some patients were lost during the follow-up, leading to a bias in the results analysis. We consider that membranous nephropathy complicated with FSGS is still an important prognostic factor in combination with the above pathological and clinical comparisons.

To date, the potential mechanism of FSGS lesions in IMN remains unclear. Most studies suggest that primary and secondary focal segmental glomerulosclerosis is thought to be caused by intrarenal vasodilation, increased glomerular capillary pressure, and structural and functional adaptation mediated by plasma flow velocity. The podocytes of the glomeruli are active cells with mechanical sensors that respond to positional stimulation and shear stress. Due to compensatory effects, the residual glomerular volume and surface area increase, exerting mechanical stress on the podocytes and causing podocyte damage, which is probably the mechanism of membranous nephropathy with focal segmental sclerosis [23]. Michio Nagata recently found that segmental glomerulosclerosis in FSGS seems to be based on the focal glomerular response to halt protein leakage from the GBM after podocyte loss, functioning as a wound healing process [24]. However, Alain Meyrier observed that some cases of secondary FSGS improved after the use of immunosuppressive agents, which may be lateral confirmation that this disease is related to immune factors [25]. In our study, our two pathologists were unable to distinguish the pathological changes of endothelial hyperplasia and foot process fusion at the FSGS site under a light microscope, while electron microscopy could not render the sclerotic globules. Therefore, it was impossible to further determine whether these sclerotic globules were secondary factors or primary factors. However, upon analysis of the relevant literature and the patients’ medical history, we discovered that most of these patients had focal segmental lesions caused by various secondary factors such as hypertension.

Of course, there are still some limitations in our research. First, this was a single-center retrospective clinical study. Second, the observation time was short, and there was a certain degree of loss to follow-up, which may cause deviation of the final data. Therefore, further large-scale clinical and basic research is needed to further confirm these results.

## Conclusion

The occurrence of segmental glomerulosclerosis can occur in the course of membranous nephropathy. Pathological changes, such a manifestation of chronic kidney injury, often indicate a poor prognosis of membranous nephropathy. Membranous nephropathy complicated with FSGS is a risk factor for IMN, but it is not an independent risk factor. Patients with membranous nephropathy complicated with FSGS often present hypertension and tubule injury; therefore, when selecting immunosuppressants, nonselective cyclophosphamide should be the first choice, and CNIs should be used cautiously.

## Data Availability

The datasets used and/or analyzed during the current study will be available from the corresponding author on reasonable request.

## Abbreviations

ALB: Albumin
ALT: Glutamic pyruvic transaminase
ANA: Antinuclear antibody
ANCA: Andante neutrophil cytoplasmic antibody
AST: Glutamic oxaloacetic transaminase
CNIs: Calmodulin neurophosphatase inhibitors
CR: Complete remission
ESRD: End-stage renal disease
FSGS: Focal segmental glomerulosclerosis
GLB: Globulin
IMN: Idiopathic membranous nephropathy
IMN-FSGS: Membranous nephropathy complicated with FSGS lesions
NFAT: Nuclear factors in activated T cells
PLA2R: Phospholipase A2 receptors;
PR: Partial remission
SLE: Systemic lupus erythematosus
SOD: Superoxide dismutase
TP: Serum total protein
TRPC6: Transient receptor potential cation channel 6 CaN Calmodulin neurophosphatase
u-NAG: N-acetyl-β-D-glucosidase
u-RBP: retinol-binding protein
